# A new formula to calculate the resection limit in hepatectomy based on Gd-EOB-DTPA-enhanced magnetic resonance imaging

**DOI:** 10.1371/journal.pone.0210579

**Published:** 2019-01-25

**Authors:** Shinichiro Yamada, Mitsuo Shimada, Yuji Morine, Satoru Imura, Tetsuya Ikemoto, Yu Saito, Chie Takasu, Masato Yoshikawa, Hiroki Teraoku, Toshiaki Yoshimoto

**Affiliations:** The Department of Surgery, Tokushima University, Tokushima, Japan; Indiana University, UNITED STATES

## Abstract

**Background and aim:**

Dynamic magnetic resonance imaging with gadolinium-ethoxybenzyl-diethylenetriamine pentaacetic acid (EOB-MRI) can be used not only to detect liver tumors but also to estimate liver function. The aim of this study was to establish a new EOB-MRI-based formula to determine the resection limit in patients undergoing hepatectomy.

**Methods:**

Twenty-eight patients with a normal liver (NL group) and five with an unresectable cirrhotic liver (UL group) who underwent EOB-MRI were included. Standardized liver function (SLF) was calculated based on the signal intensity (SI), the volume of each subsegment (S1–S8), and body surface area. A formula defining the resection limit was devised based on the difference in the SLF values of patients in the NL and UL groups. The formula was validated in 50 patients who underwent EOB-MRI and hepatectomy.

**Results:**

The average SLF value in the NL and UL groups was 2038 and 962 FV/m^2^, respectively. The difference (1076 FV/m^2^) was consistent with a 70% in resection volume. Thus, the resection limit for hepatectomy was calculated as a proportion of 70%: 70×(SLF−962)/1076 (%). The one patient who underwent hepatectomy over the resection limit died due to liver failure. In other 49 patients, in whom the resection volume was less than the resection limit, procedures were safely performed.

**Conclusions:**

Our formula for resection limit based on EOB-MRI can improve the safety of hepatectomy.

## Introduction

In spite of improvement in the outcome of liver surgery during the past few decades, post-operative liver failure remains a serious complication and is currently the leading cause of post-operative mortality [1–3]. Therefore, the assessment of hepatic functional reserve is one of the most important issues in liver surgery. This is especially true for patients with hepatocellular carcinoma (HCC) and underlying chronic liver disease. Although no “gold standard” has been established, several studies have demonstrated the value of preoperative liver function tests, such as indocyanine green dye retention at 15 min (ICGR15) and volumetric assessments of the liver using Tc-99m galactosyl human serum albumin (GSA) [4–9], to estimate hepatic functional reserve in patients scheduled for hepatectomy. However, there are several problems in evaluating liver function with these tests. Because the ICGR15 estimates only total liver function, it may fail to detect regional dysfunction or hepatic compensation of regional defects [10]. ICGR15 is also affected by high bilirubinemia and intrahepatic shunt [11]. GSA is a receptor binding agent specific for the asialoglycoprotein receptor expressed exclusively on the plasma membrane of hepatocytes. The tracer is bound only by this receptor and thus provides valuable information about receptor population density, which also directly reflects the amount of functioning hepatocyte mass [12]. However, despite the reported utility of GSA in estimating liver function [13], it may underestimates left lobe function [14], and this test involves radioactive isotope injection and thus carries certain risks as well as costs [15].

Gadolinium-ethoxybenzyl-diethylenetriamine pentaacetic acid (Gd-EOB-DTPA) is a magnetic resonance imaging (MRI) contrast medium used to examine the hepatobiliary system. As it is lipophilic, Gd-EOB-DTPA is easily taken up by hepatocytes and secreted into the biliary system without any change in its chemical structure [16]. Consequently, dynamic MRI with Gd-EOB-DTPA (EOB-MRI) can be used to quantitatively evaluate liver function, although the method was originally developed to improve the detection of small hepatic tumors [17, 18]. Recently, several studies have confirmed a correlation between liver function and various imaging parameters obtained using EOB-MRI [19–21].

In previous work, our group determined the correlation coefficients between the relative signal intensity (SI) values of EOB-MRI and preoperative liver function, as assessed using GSA, ICGR15, and the prothrombin time [13]. Furthermore, EOB-MRI can be used in the clinical setting to estimate regional liver functional reserve, such as the case of hilar cholangiocarcinoma showing the difference in liver function between the right and left lobes [13]. Accordingly, EOB-MRI may compensate for the limitations of ICGR15 and GSA.

In recent years, hepatectomy has been underwent based on the decision tree so called “Makuuchi criteria,” widely used in Japan and other Asian countries. The criteria takes into account the presence or absence of ascites, the serum total bilirubin level, and the ICGR15 value [22]. The safety limit of the hepatic resection rate can be estimated using this criteria for selecting patients and hepatectomy procedures have been proposed [23]. However, as mentioned above, EOB-MRI may provide a more accurate assessment of liver function than obtained with ICGR15 and GSA. Demonstration of the ability of EOB-MRI to establish the new resection limit while detecting the tumor and estimating liver function would obviate many preoperative assessment procedures. Therefore, the purpose of this study was to devise a new formula for the resection limit in hepatectomy patients using SI values obtained by EOB-MRI (EOB-SI).

## Patients and methods

### Patients and imaging

Patients with normal liver function as well as those with impaired function due to unresectable liver disease were selected to devise a formula for determining the resection limit. The normal liver function (NL) group comprised 28 patients, including 2 who suffered from HCC without viral infection and had a normal ICGR15 (<10%), 23 patients with metastatic liver cancer who had not been treated with any form of chemotherapy and had a normal ICGR15, and 3 patients who were living donors for liver transplantation. In the unresectable (UL) group were 5 liver transplant recipients with impaired liver function and massive ascites.

All 33 patients underwent MRI using a superconducting magnet operating at 1.5 T (Signa EXCITE HD, GE Medical Systems, Milwaukee, WI, USA) and an 8-channel phased-array coil. As described in detail previously [13], Pre-contrast T1-weighted fast spoiled gradient echo imaging was followed by T2-weighted spin echo and diffusion-weighted single-shot spin-echo echo-planar imaging. Dynamic images using fat-suppressed T1-weighted gradient-echo images with a three-dimensional (3D) acquisition sequence (LAVA: liver acquisition with volume acceleration) were obtained before (precontrast) and 20 s, 60 s, 2 min, 5 min, 10 min, and 20 min after the intravenous administration of 0.1 mg Gd-EOB-DTPA /kg body weight using a power injector (Sonic Shot 50, Nemoto, Tokyo, Japan). The tracer was administered as a bolus dose at a rate of 3 mL/s through an intravenous cubital line (20–22 gauge) that was subsequently flushed with 20 mL of saline, also using a power injector. Hepatocyte-phase images obtained 20 min post-injection were used in this study.

This study was approved by the Institutional Review Board of Tokushima University Hospital (No. 2478), and patients provided informed written consent to have data from their medical records used in this study.

### Image analysis in EOB-MRI

As described in our previous report [13], the SI values of the eight Couinaud subsegments of the liver were measured using a circular region of interest (ROI) of approximately 100 mm^2^. In each subsegment of the liver, the SIs of three ROIs were measured and the average then calculated. The SI of the erector spine muscle at the level of the porta hepatis was also determined. The relative signal intensity for each segment was calculated as follows: relative SI = SI of each eight Couinaud subsegments (from S1 to S8) / SI of muscle. Since all eight liver segments were evaluated, patients with large liver tumors were excluded from the study.

### Definition of standardized liver function (SLF)

**Fig 1:**
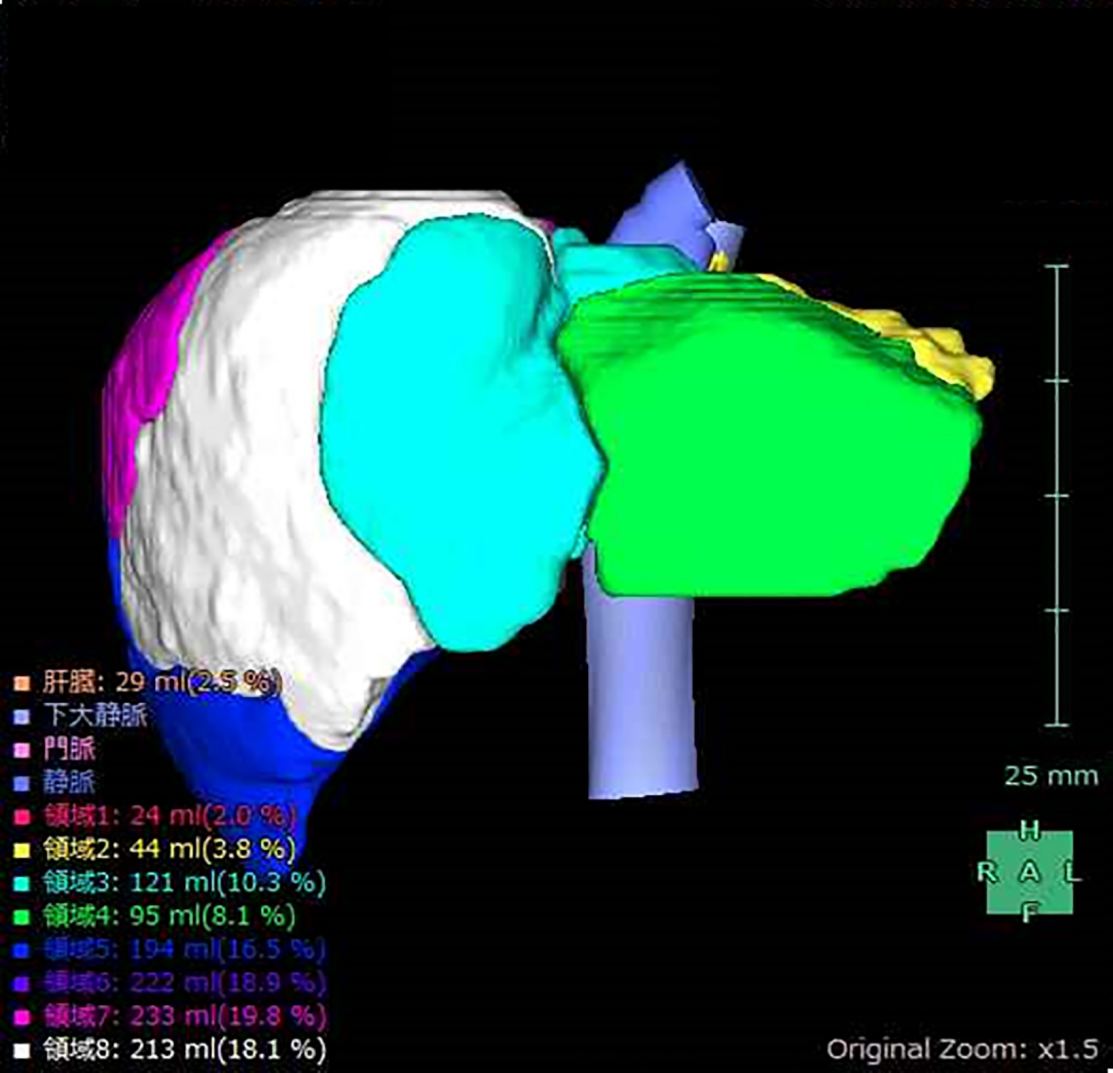
Volume calculation using SYNAPSE VINCENT. Simple volume of each liver subsegment (S1–S8), calculated using the 3D volume analyzer SYNAPSE VINCENT.

The simple volume of each segment (S1–S8) was calculated using the 3D volume analyzer SYNAPSE VINCENT (Fujifilm Medical, Tokyo, Japan) (Fig 1). The functional volume (FV) was calculated by multiplying the relative SI by the volume of each subsegment. The total FV (S1-8) was then divided by the body surface area of the patient; this value was defined as the standardized liver function (SLF), expressed as FV/m^2^.

$$SLF=\frac{\sum_{n=1}^{8}relative\;SI\times volume(Sn)}{Body\;surface\;area}\;(FV/m2)$$

A formula to determine the resection limit was devised based on the difference between the SLF of the NL group and that of the UL group (see the Results section).

### Validation of the hepatic resection limit formula using SI

The utility of the formula was confirmed in 50 patients who underwent hepatectomy and preoperative EOB-MRI.

## Results

### Patient characteristics and SLF values

**Fig 2:**
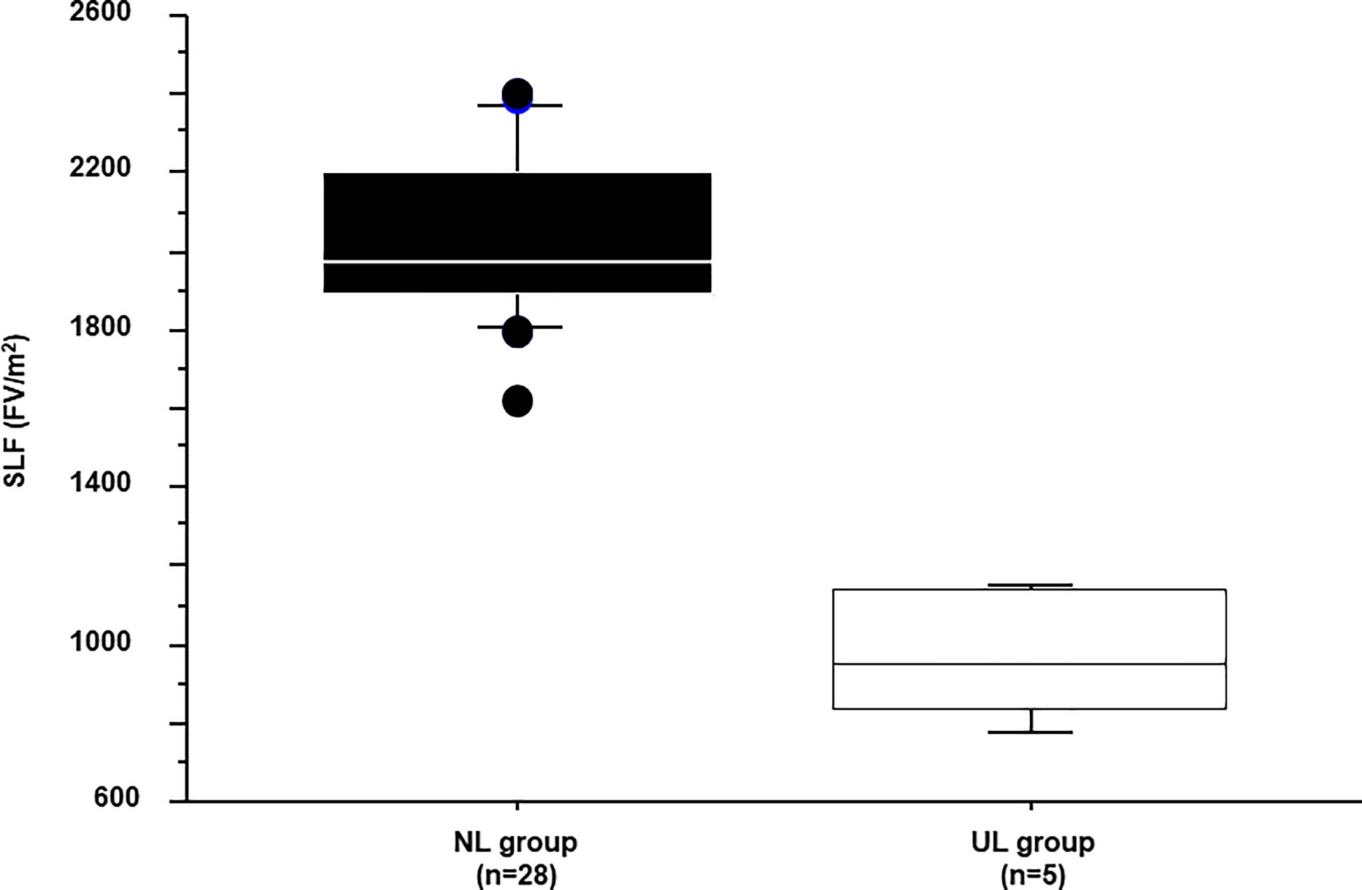
Standardized liver function. Standardized liver function **(**SLF) in patients with a normal liver (NL group) and those with an unresectable liver (UL group). The average values in the two groups were 2038 and 962 (FV/m^2^), respectively.

**Fig 3:**
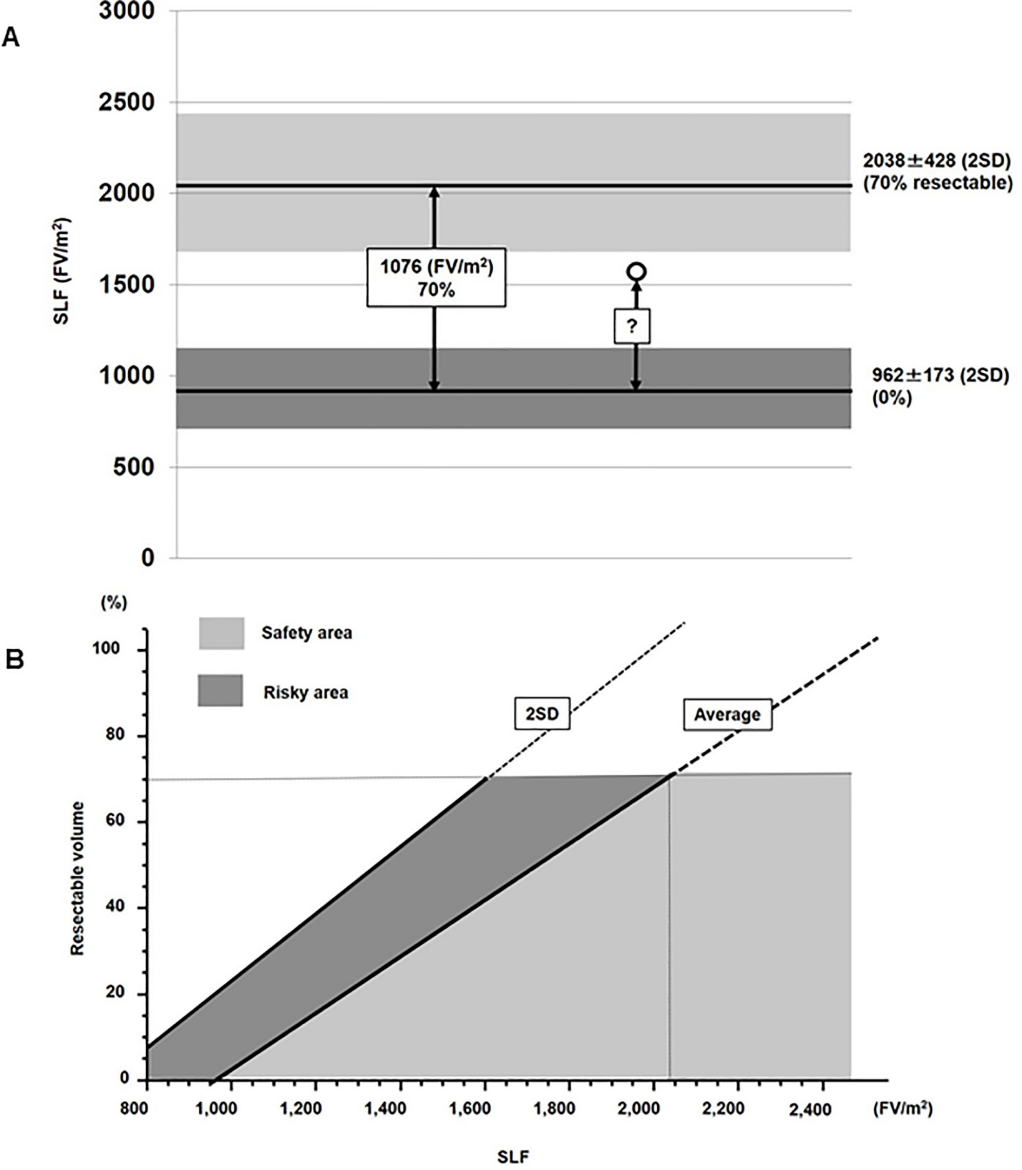
**(A) Calculation of the resection limit.** The average SLF of the NL group (2038) was set as 70% resectable, and that of the UL group (962) as 0% resectable. The liver resection limit for patients undergoing hepatectomy (white circles) was calculated based on a SLF of 70%. **(B) Resection limit calculated from the SLF.** Both an average line and a 2SD line are displayed. The average line defines the “safety limit”, and the 2SD line the “maximum limit.” Accordingly, the area under the average line (light gray) is the “safety area”, and that between the 2SD line and the average line (dark gray) the “risk area”.

The characteristics of the 33 patients are shown in Table 1, and their SLF values in Fig 2. The average SLF±2SD of the NL and UL group were 2038±428 and 962±173 (FV/m^2^), respectively. Since patients with a normal liver can tolerate a 60%–80% hepatectomy [24], the average SLF of the NL group (2038 FV/m^2^) was set as 70% resectable, and that of the UL group (962 FV/m^2^) as 0% resectable. Therefore, the difference between the NL and UL groups (1076 FV/m^2^) corresponded to a resection volume of 70%, which was then used to calculate the resection limit for patients undergoing hepatectomy (Fig 3A).

**Table 1:** Characteristics of the patients in the normal liver (NL) and unresectable liver (UL) groups.

Variable	NL (n = 28)	UL (n = 5)
Age (y)	61.2±11.6	54.2±10.0
Sex (M/F)	20/8	4/1
Hepatitis type (B/C/NBNC)	0/0/28	0/1/3
Child classification (A/B/C)	28/0/0	0/2/3
Albumin (g/dL)	4.1±0.3	3.2±1.1
Total bilirubin (mg/dL)	0.7±0.2	6.4±4.4
AST (U/L)	22.0±7.9	84.0±78.5
Platelets (×104/μL)	27.5±11.0	7.8±4.7
ICGR15 (%)	6.1±2.5	43.5±14.2
Prothrombin time (%)	108.0±22.6	43.0±14.2
Type IV collagen (ng/ml)	147.2±58.3	406.0±98.1
Hyaluronic acid (ng/ml)	59.8±47.9	688.6±287.9

AST: aspartate aminotransferase; ICGR15: indocyanine green retention in 15 min. All data are expressed as the mean ± SD.

### Formula for the determination of the resection limit

The formula for the determination of the resection limit was therefore 70×(SLF-962)/1076 (%) and is depicted as an average line in Fig 3B, together with a 2SD line. The average line can be considered as the “safety limit” and the 2SD line as the “maximum limit.” Thus, the area under the average line (light gray) is the “safety area” and that between the 2SD and average lines (dark gray) as the “risky area.”

### Validation of the formula using EOB-MRI and the SI

**Fig 4:**
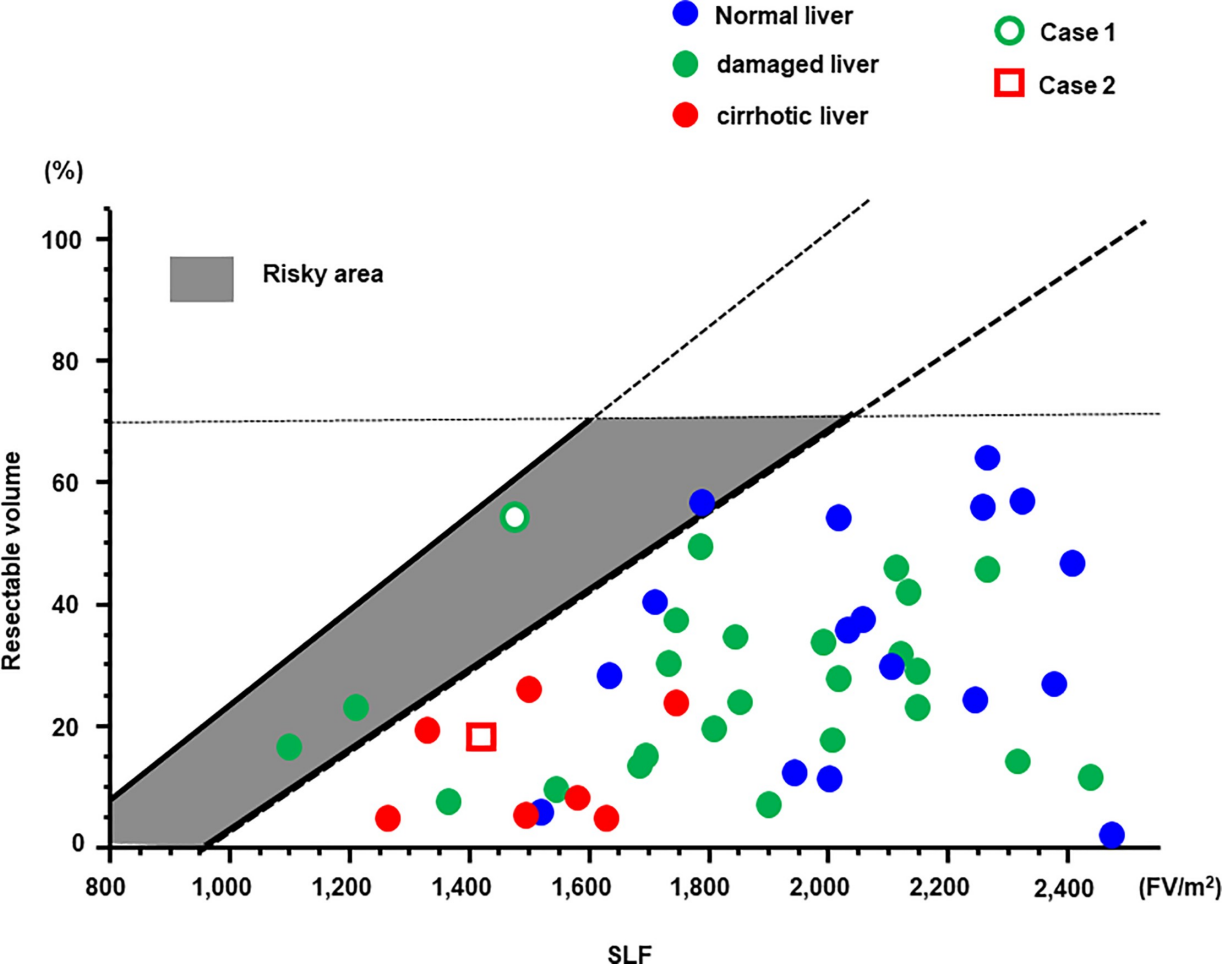
Resection limit formula in validation set. The SLF and the resected liver volume in the 50 patients included in the validation of the resection limit formula.

The formula for the determination of the resection limit was validated in 50 patients who underwent EOB-MRI prior to hepatectomy. Their characteristics are shown in Table 2, and their SLF and resected liver volumes in Fig 4. Eight patients, represented as red color, showed liver cirrhosis due to viral hepatitis, Alcoholic or non-alcoholic steatohepatitis (NASH). Twenty-five patients, represented as green color, had damaged liver, but not cirrhotic, due to such as viral hepatitis, NASH or chemotherapy. Seventeen patients, represented as blue color, had normal liver. All cirrhotic patients were planed liver resection in safety area. Only one patient who underwent hepatectomy over the safety limit died due to postoperative liver failure (white circle). Of the remaining 46 patients who underwent hepatectomy within the safety area and 3 patients in risky area, none suffered liver failure. Seven patients had complications worse than Clavien-Dindo III [25], such as bile leakage and pneumonia. These patients showed longer postoperative hospital stay than other patients without complications, and start of adjuvant chemotherapies were delayed in 2 patients. However, these complications did not affect on liver function because liver regeneration of these patients assessed by postoperative CT was not inhibited. Two representative cases are presented in the following.

**Table 2:** Characteristics of the 50 patients in the validation set.

Variable	n = 50
Age (y)	66.0±14.4
Sex (M/F)	33/17
Diagnosis (HCC/meta/IHCC/Other)	20/15/6/9
Hepatitis type (B/C/NBNC)	11/20/19
Child classification (A/B)	50/0
Albumin (g/dL)	3.7±0.6
Total bilirubin (mg/dL)	0.8±0.4
AST (U/L)	37.2±25.3
Platelets (×104/μL)	21.4±11.9
ICGR15 (%)	11.4±7.3
Prothrombin time (%)	104.8±22.3
Type IV collagen (ng/ml)	154.6±88.2
Hyaluronic acid (ng/ml)	116.9±156.2

HCC: Hepatocellular carcinoma; IHCC: Intrahepatic cholangiocarcinoma. All data are expressed as the mean ± SD.

#### Case 1

**Fig 5:**
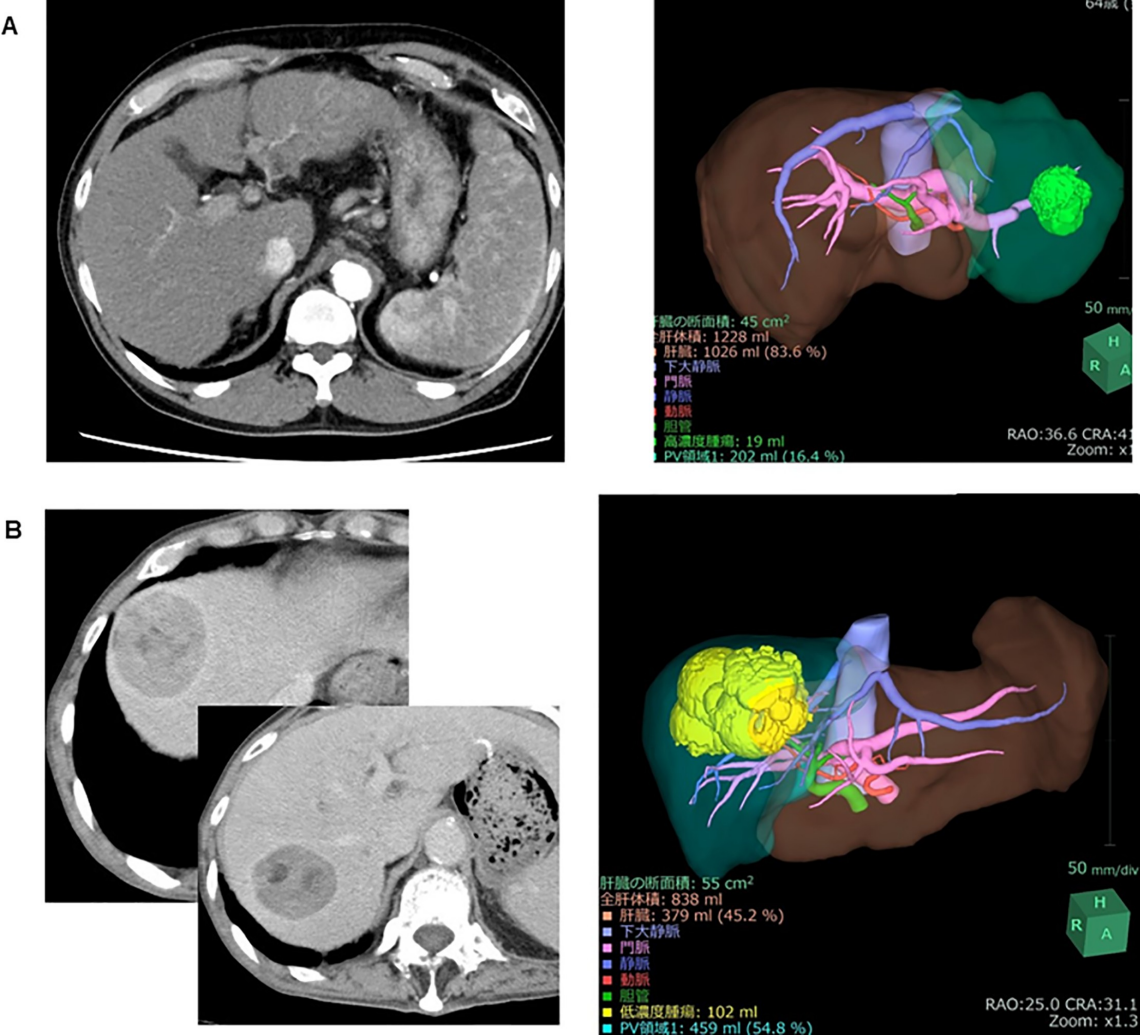
**(A) Representative cases in the validation set.** Abdominal enhanced computed tomography (CT) shows a very large tumor in the right lobe. Based on 3D volumetry using SYNAPSE VINCENT, the remnant volume after right lobectomy of the liver was 45.2%. **(B) Representative cases in the validation set.** Abdominal enhanced CT shows the tumor in the lateral segment (arrowhead). On 3D volumetry using SYNAPSE VINCENT, the volume of the lateral segment was 16.4%.

A male patient in his 80s who was diagnosed with HCC was admitted to our hospital. He suffered from type C hepatitis and his ICGR15 was 15%. The very large tumor necessitated right lobectomy of the liver to achieve curative resection. Although a left lobectomy or segmentectomy were recommended by the Makuuchi criteria, 3D simulation using VINCENT showed that remnant liver volume after right lobectomy was almost 50% because tumor was large (Fig 5A). Therefore, right lobectomy was performed. Unfortunately, this patient died due to liver failure 8 days postoperatively. The safety limit and maximum limit in this case were 35% and 58%, respectively; thus, this case was located in risky area. The advanced age of the patient and the relatively large (826 ml) intraoperative blood loss might be additional factors that contributed to his death.

#### Case 2

A male patient in his 60s was admitted to our hospital with intrahepatic chorangiocarcinoma. Because the tumor was located in liver segments S2 and S3, lateral segmentectmy of the liver was necessary to achieve curative resection. The patient suffered from alcoholic liver cirrhosis; his ICGR15 value was 21.9%. Based on the Makuuchi criteria, a subsegmentectomy was recommended. However, 3D simulation using VINCENT showed that the volume of the lateral segment was 16.4% (Fig 5B) as the safety limit was 30%. Lateral segmentectomy of the liver was safely performed without postoperative complications.

## Discussion

EOB-MRI is highly sensitive for detecting small hepatic nodules [17, 18], and has recently become one of the standard preoperative imaging modalities for patients with liver tumors. In addition, several recent studies have also suggested the use of EOB-MRI to estimate liver function [26–30]. This finding is supported by our own work, which has shown that EOB-MRI can be used to estimate liver function in a clinical setting [13].

To our knowledge, this study is the first to determine the resection limit using EOB-MRI. Specifically, we devised a formula to establish the resection limit, using the SLF values calculated from the relative SI, as determined by EOB-MRI. Although some patients underwent procedure over Makuuchi criteria, all 46 patients in safety area and 3 patients in risky area underwent hepatectomy safely. By contrast, the one patient in risky area died due to postoperative liver failure. This patient was over 80 years old and had relatively large intraoperative blood loss. Other 3 patients in risky area were not so old (under 75 years old), and surgical invasiveness was relatively small from the viewpoint of operative time and blood loss. In patients with a resection limit falling within the risky area extra caution must therefore be taken not to induce liver failure, with respect to not only intraoperative procedures but also perioperative complications and risk factors such as advanced age. Seven patients had post-operative complications such as bile leakage or pneumonia, which seemed to have small relationship with liver function.

Advantage of EOB-MRI in this study is that comprehensive preoperative information regarding hepatectomy, predicting resection limit in addition to detecting small liver tumors and estimating liver function, can be obtained in a single imaging study. Besides the convenience and time savings, this approach also leads to cost reductions.

Moreover, the use of EOB-MRI to estimate regional liver function has been reported. Xiao et al. showed that EOB-MRI is an effective modality for evaluating the impairment of regional liver function following TACE therapy, based on the signal-to-noise ratio [31]. Henlik, et al. have insisted the non-homogeneous distribution of liver function in cirrhotic patients, and regional function assessment by EOB-MRI could minimize the risk of postoperative liver failure and possibly death [32]. Ninomiya et al. reported the use of EOB-MRI to estimate regional liver function after liver transplantation [33].

Although there was no major difference in the regional liver function of the 50 patients who participated in a validation of the restriction limit formula in this study, in some of other patients liver function between Couinaud segments varied. S1 Fig shows the case with cholangiocarcinoma in S8. In this segment, the peripheral bile duct was dilated due to the tumor and the relative SI was lower than in other segments. This result shows that EOB-MRI can estimate liver function at the subsegmental level. The findings in a patient with hilar cholangiocarcinoma are presented in S2 A and S2B Fig. Severe atrophy in the right lobe of the liver due to bile duct obstruction by the tumor was detected, consistent with the major difference in the SI values of the right and left lobes. The FV, determined by multiplying the relative SI by the simple volume of each subsegment, is shown in S2C Fig. Although the simple volume of the right lobe was 34.1% of the total liver volume, the FV of the right lobe based on the relative SI was 25.7%. In this patient, the safety limit for resection was 33.2%, and right lobectomy needs slightly larger volume than resection limit (risky area) by means of simple volume. (S2D Fig). However, according to the FV, the resection volume was located within the safety area. This case underwent right lobectomy of the liver safely, and demonstrated the utility of EOB-MRI in estimating the safety of hepatectomy, even in patients with regional differences in liver function. This is in contrast to global liver function tests, including ICGR15. While the use of GSA to estimate regional liver function has also been reported, the examination is not yet widely available. Moreover, GSA may underestimate left lobe function, due to partial volume effects and tracer accumulation in the heart [14]. EOB-MRI has an advantage over GSA in that it accurately estimates liver function in addition to detecting liver tumors.

The present study has several limitations. Firstly, calculation of the SLF was based on a small number of patients. Particularly, in the UL group, determination of the SLF would have benefited from the inclusion of a larger number of patients. Secondly, in patients with a small liver volume the SLF tended to be lower, despite the correction by body surface area. This also remains to be verified with a larger number patients.

In conclusion, our new formula of resection limit using EOB-SI is useful for safe hepatectomy. Surgeons can obtain many important information regarding tumor, liver function and resection limit in this single imaging study.

## Supporting information

S1 Fig Case of cholangiocarcinoma in S8. (A) The axial view shows the location of the tumor, at S8. Within the segment, the peripheral bile duct was dilated (arrowhead). (B) On a coronal view, the signal intensity (SI) was lower in S8 than in other segments.
(TIF)

S2 Fig Case of hilar cholangiocarcinoma. (A) Abdominal enhanced CT shows atrophy of the right lobe of the liver due to the tumor, located at the hilum. (B) On hepatobiliary phase EOB-MRI, the SI of the right lobe is lower than that of the left lobe. (C) Regional liver function in the right lobe, calculated as the relative SI × simple volume of each subsegment, was 25.7%.
(D) Resection limit. The safety limit was 33.2% but the right lobectomy volume needed to achieve cure was slightly larger than the resection limit (white circle) determined based on the simple volume. However, when regional liver function was calculated based on the relative SI, the resection volume was located in the safety area (black circle).
(TIF)
