# Supplementary figures and images for: A new formula to calculate the resection limit in hepatectomy based on Gd-EOB-DTPA-enhanced magnetic resonance imaging
Source: PLoS One. 2019 Jan 25;14(1):e0210579. doi: 10.1371/journal.pone.0210579 (PMC6347147; doi:10.1371/journal.pone.0210579)

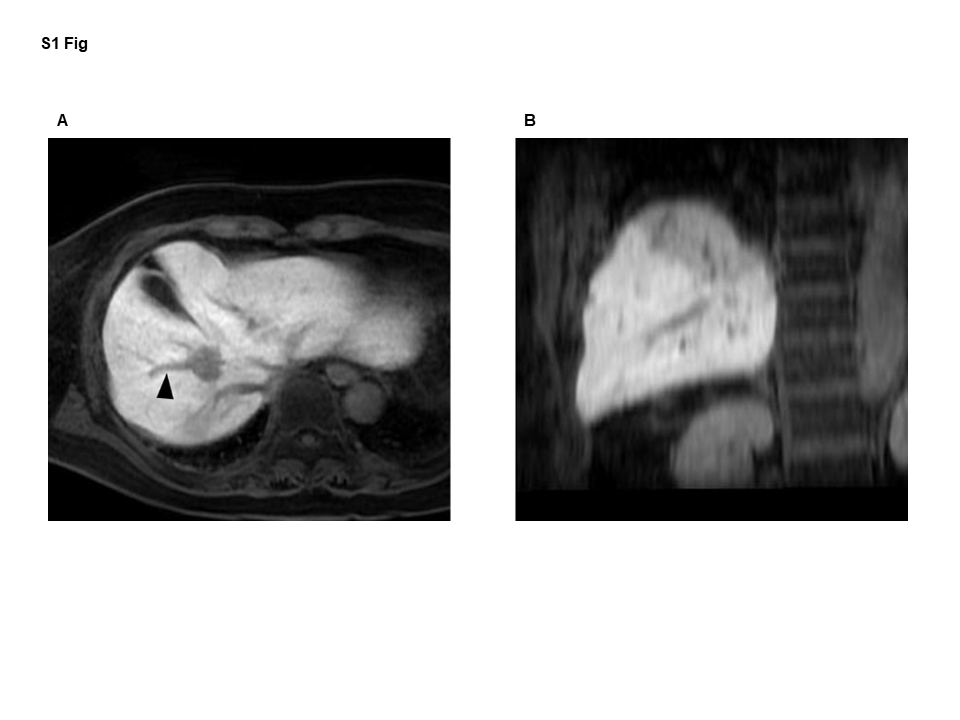

Supplement: S1 Fig — (A) The axial view shows the location of the tumor, at S8. Within the segment, the peripheral bile duct was dilated (arrowhead). (B) On a coronal view, the signal intensity (SI) was lower in S8 than in other segments. (TIF) [file pone.0210579.s001.TIF]

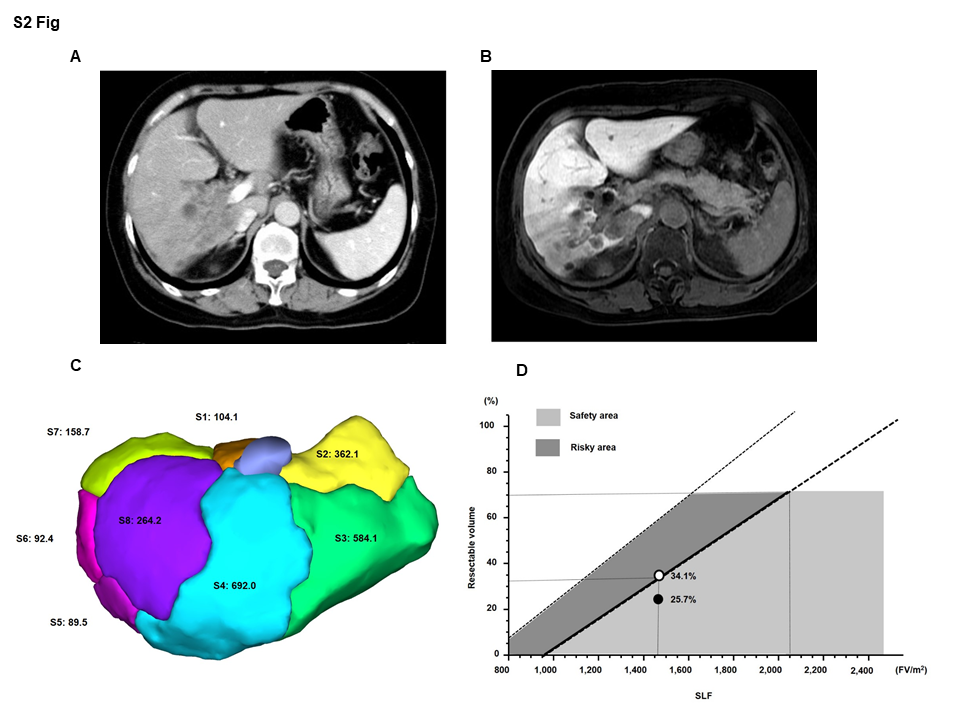

Supplement: S2 Fig — (A) Abdominal enhanced CT shows atrophy of the right lobe of the liver due to the tumor, located at the hilum. (B) On hepatobiliary phase EOB-MRI, the SI of the right lobe is lower than that of the left lobe. (C) Regional liver function in the right lobe, calculated as the relative SI × simple volume of each subsegment, was 25.7%. (D) Resection limit. The safety limit was 33.2% but the right lobectomy volume needed to achieve cure was slightly larger than the resection limit (white circle) determined based on the simple volume. However, when regional liver function was calculated based on the relative SI, the resection volume was located in the safety area (black circle). (TIF) [file pone.0210579.s002.TIF]
